# Different ODE models of tumor growth can deliver similar results

**DOI:** 10.1186/s12885-020-6703-0

**Published:** 2020-03-17

**Authors:** James A. Koziol, Theresa J. Falls, Jan E. Schnitzer

**Affiliations:** Proteogenomics Research Institute for Systems Medicine (PRISM), La Jolla, California 92037 USA

**Keywords:** Tumor growth, Cancer chemotherapy, Mathematical model, Ordinary differential equations, Delay differential equations

## Abstract

**Background:**

Simeoni and colleagues introduced a compartmental model for tumor growth that has proved quite successful in modeling experimental therapeutic regimens in oncology. The model is based on a system of ordinary differential equations (ODEs), and accommodates a lag in therapeutic action through delay compartments. There is some ambiguity in the appropriate number of delay compartments, which we examine in this note.

**Methods:**

We devised an explicit delay differential equation model that reflects the main features of the Simeoni ODE model. We evaluated the original Simeoni model and this adaptation with a sample data set of mammary tumor growth in the FVB/N-Tg(MMTVneu)202Mul/J mouse model.

**Results:**

The experimental data evinced tumor growth heterogeneity and inter-individual diversity in response, which could be accommodated statistically through mixed models. We found little difference in goodness of fit between the original Simeoni model and the delay differential equation model relative to the sample data set.

**Conclusions:**

One should exercise caution if asserting a particular mathematical model uniquely characterizes tumor growth curve data. The Simeoni ODE model of tumor growth is not unique in that alternative models can provide equivalent representations of tumor growth.

## Background

Simeoni and colleagues [1–3] introduced and developed a pharmacokinetic/pharmacodynamic (PK/PD) model of tumor growth from in vivo animal studies, which could be summarized mathematically by a system of ordinary differential equations (ODEs). The model, and adaptations, have proved quite successful in predicting or modeling tumor growth and efficacy of cancer treatments [4–9].

A distinguishing characteristic of the Simeoni tumor growth model is that under chemotherapy, a drug’s action is not instantaneous: rather, tumor cells pass through progressive stages of damage because of the drug’s mechanism of action. This delay in drug action is modeled through a series of delay compartments through which the cells transit before elimination. In this note, we propose an alternative mathematical formulation of the Simeoni model using delay differential equations, without recourse to a series of delay compartments. We describe the two models in the next section, and then compare their performances with experimental data relating to mammary tumor growth in a mouse model. We conclude that one should be cautious if asserting that a particular tumor growth model uniquely characterizes experimental data.

## Methods

### The Simeoni model

**Fig. 1:**
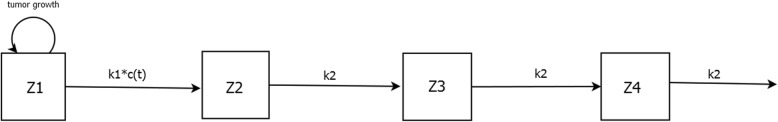
Schematic representation of the Simeoni tumor growth model. The tumor resides in compartment Z_1_, with growth described by a tumor growth function. c(t) denotes the plasma concentration of an anticancer agent if present. The drug elicits its effect decreasing the tumor growth rate by a factor proportional to c(t)*Z_1_(t) through the constant parameter k_1_. Tumor cells cycle successively through transit compartments Z_2_, Z_3_, Z_4_ before cell death. k_2_ is a first-order rate constant of transit. The number of transit compartments is arbitrary. The system of ordinary differential equations describing this model is given in the text

The Simeoni model is depicted in Fig. 1. The central compartment Z_1_ represents the actively growing tumor, which increases according to a growth function TGF. c(t) denotes the concentration of a chemotherapeutic or immunotherapeutic agent in the central compartment Z_1_ at time t. In turn, c(t) induces a fraction of tumor cells to commit to cell death with a killing constant k_1_. Tumor cells damaged by pharmacological treatment are shunted off successively into the peripheral compartments Z_2_, Z_3_, Z_4_ with a rate constant k_2_, followed by elimination representing cell death. The observed tumor volume is the sum of cells in compartments Z_1_, Z_2_, Z_3_, Z_4_. The system of differential equations prescribing the Simeoni model is as follows:

1 $$ {\displaystyle \begin{array}{l}\frac{d{Z}_1(t)}{dt}= TGF(t)-{k}_1c(t){Z}_1(t)\\ {}\frac{d{Z}_2(t)}{dt}={k}_1c(t){Z}_1(t)-{k}_2{Z}_2(t)\\ {}\frac{d{Z}_3(t)}{dt}={k}_2{Z}_2(t)-{k}_2{Z}_3(t)\\ {}\frac{d{Z}_4(t)}{dt}={k}_2{Z}_3(t)-{k}_2{Z}_4(t)\end{array}} $$

with initial conditions *Z*_1_(0) = *V*_0_, *Z*_2_(0) = *Z*_3_(0) = *Z*_4_(0) = 0. Total tumor volume is

2 $$ V(t)={Z}_1(t)+{Z}_2(t)+{Z}_3(t)+{Z}_4(t), $$

and the tumor growth function TGF(t) is given by

3 $$ TGF(t)=\frac{\lambda_0{Z}_1(t)}{{\left[1+{\left(\frac{\lambda_0}{\lambda_1}V(t)\right)}^{\psi}\right]}^{1/\psi }} $$

Although we have depicted the Simeoni model with 3 peripheral compartments, we note that the number of peripheral compartments is in general arbitrary.

**Fig. 2:**
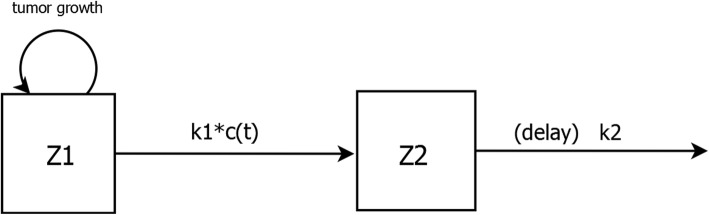
Schematic representation of the Simeoni tumor growth model, with transit compartments replaced by a single compartment in which tumor cell death is delayed relative to drug treatment. The delay is explicitly incorporated into the system of ordinary differential equations describing this model

The model incorporating a delay differential equation is conceptually similar to the Simeoni model, the only difference being that the compartments Z_2_, Z_3_, and Z_4_ are replaced by a single compartment that incorporates a delay in elimination (Fig. 2). The system of differential equations describing this model is:

4 $$ \frac{d{Z}_1(t)}{dt}= TGF(t)-{k}_1c(t){Z}_1(t) $$

$$ \frac{d{Z}_2(t)}{dt}={k}_1c(t){Z}_1(t)-{k}_2 delay\left({Z}_2,{t}_2\right), $$

where

$$ delay\left({Z}_2,{t}_2\right)={Z}_2\left(t-{t}_2\right), $$

TGF(t) and c(t) are as before, V(t) = Z_1_(t) + Z_2_(t), and initial conditions are Z_1_(0) = V_0_, Z_2_(0) = 0, and Z_2_(t) = 0 for t < 0. The variable t_2_ represents the time delay before elimination.

In the absence of chemotherapeutic or immunotherapeutic intervention (i.e., c(t) = 0), tumor growth in the Simeoni formulation reduces to the biphasic process TGF(t), characterized by initial exponential growth followed by linear growth. In particular, in contrast to more classic models of tumor growth, e.g. logistic, Gompertz, Von Bertalanffy, there is no plateau or upper limit of tumor size. One of these alternative models might credibly be more appropriate than TGF in certain experimental settings. The generalized logistic model is defined by

5 $$ {\displaystyle \begin{array}{l}\frac{dV}{dt}= aV\left(1-{\left(\frac{V}{K}\right)}^{\nu}\right)\\ {}V(0)={V}_0,\end{array}} $$

the Gompertz model is given by

6 $$ {\displaystyle \begin{array}{l}\frac{dV}{dt}= aVln\left(\beta V\right)\\ {}V(0)={V}_0,\end{array}} $$

and the Von Bertalanffy model is

7 $$ {\displaystyle \begin{array}{l}\frac{dV}{dt}=a{V}^{\gamma }- bV\\ {}V(0)={V}_0.\end{array}} $$

These three models have analytic solutions [10], which might aid in nonlinear fitting to experimental data.

### In vivo tumor growth experiments

A series of experiments was undertaken at PRISM, the goal being to establish a viable and reproducible mouse model for mammary tumors. The FVB/N-Tg(MMTVneu)202Mul/J model was used for this purpose (Jackson stock #002376). All mice were bred at PRISM, with female mice genotyped by polymerase chain reaction to confirm transgene expression. Focal mammary tumors appear in female mice at about 100 days, reset as day 0 to mark the onset of the experiment. Data from one particular experiment are considered in the present work. In this experiment, 40 mice were randomly assigned to either no treatment (*n* = 21) or a single dose of cisplatin (5 mg/kg, *n* = 19) on day 0; all animals were relatively healthy and functioning on day 0, with all tumors smaller than 700 mm^3^ at initiation. The primary experimental outcome consisted of the series of tumor measurements from each animal, which were taken daily (excepting weekends, holidays) by a single individual (TJF) using digital calipers, and tumor volumes were recorded using the approximating formula V = (Length x Width^2^)/2, where L > W. [In this approximating formula, tumor shape is taken as an ellipsoid generated from the rotation of a semi-ellipsis around its larger axis (length), and a multiplicative constant involving p is ignored.] Mice were euthanized when tumors reached a volume of ~ 2500 mm^3^ (or measured 17 mm in length + width + height), or for complications, e.g., interference, ulceration. The method of euthanization was CO_2_ narcosis followed by cervical dislocation, a method consistent with the 2013 recommendations of the American Veterinary Medical Association Panel on Euthanasia. The protocol for this study was approved by the Institutional Animal Care and Use Committee of Prism, confirmed by the National Institutes of Health Office of Laboratory Animal Welfare Assurance, #D16–00819. Animals were housed in Prism’s animal care facility.

All 40 animals initially randomized to the untreated and the cisplatintreated groups were included in the subsequent analyses. There were a total of 386 observations among the 21 control animals, and 545 observations among the 19 treated animals.

### Statistical methods

A nonlinear mixed effects (NLME) model was used to fit growth curves to the longitudinal tumor size data. In our context, a general form for NLME models would be:

$$ {y}_{ij}=f\left({t}_{ij},{\varphi}_i\right)+g\left({t}_{ij},{\varphi}_i,\zeta \right){\varepsilon}_{ij},\dots .1\le i\le N,\dots 1\le j\le {n}_i, $$

where N is the number of individuals, n_i_ is the number of observations for individual i, t is the regression variable time, and y are the observations (tumor volumes). The term f is the structural model, expressed from the systems of ordinary differential equations shown earlier.

The residual error is *g*(*t*_*ij*_, *φ*_*i*_, *ζ*)*ε*_*ij*_, where *ε*_*ij*_ ∼ *N*(0, *σ*^2^) In our modeling,

we chose the function *g* to be a linear combination of a constant term and a term proportional to the structural model *f* with the additional parameters *ζ* = (*a*, *b*), that is, *y* = *f* + (*a* + *b* ∗ *f*)*e.*

The individual parameters *φ*_*i*_ are defined as follows:

$$ {\varphi}_i=h\left(\mu, {\lambda}_i\right),\dots .{\lambda}_i\sim N\left(0,\varOmega \right),\dots . $$

$$ i=1,...,N, $$

where m is a p-dimensional vector of fixed population parameters, l_*i*_ is a p-vector of random effects, W is the p x p variance-covariance matrix of the random effects, and *h* is a fixed transformation. We assume that all of the model parameters are log-normally distributed among the individuals in each cohort, so that *h(x) = exp(x)*.

Parameter estimation of the typical population values and the interindividual variability of each model parameter were estimated using Monolix 2019R1 (Lixoft, Antony, France). The Monolix suite of programs implements a stochastic approximation expectation maximization (SAEM) algorithm [11, 12] for maximum likelihood estimation of the model parameters. With the treated animals, following a single bolus injection of cisplatin we took *c(t)* to represent first order elimination, that is, a constant proportion of the drug is eliminated per unit time.

Model selection and comparison was based on the Akaike information criterion (AIC), the corrected Akaike information criterion (AIC_c_), and the Bayesian information criterion (BIC). AIC_c_ is a modified version of the AIC, and includes a correction term for small sample sizes [13–15]:

8 $$ AI{C}_c= AIC+\frac{2k\left(k+1\right)}{n-k-1} $$

where k denotes the number of free parameters, and n is the number of observations. In our setting, we also included variance components from the random effects in the parameter count.

Given a set of candidate models, each with a specific IC (AIC, AIC_c_, BIC) value, we calculate IC model weights [15–17] for comparative purposes. We first compute for each model *j* the difference in IC relative to the IC of the best candidate model: D_j_ = IC_j_ - min IC. We then obtain the model weights by transforming to the likelihood scale and normalizing:

9 $$ {w}_j=\frac{\exp \left(-{\varDelta}_j/2\right)}{\sum \limits_{m=1}^M\exp \left(-{\varDelta}_m/2\right)} $$

These weights are measures of the strength of evidence. They sum to one, and reflect the probability of each model *j* given the data and the M candidate models [15, 17].

## Results

**Fig. 3:**
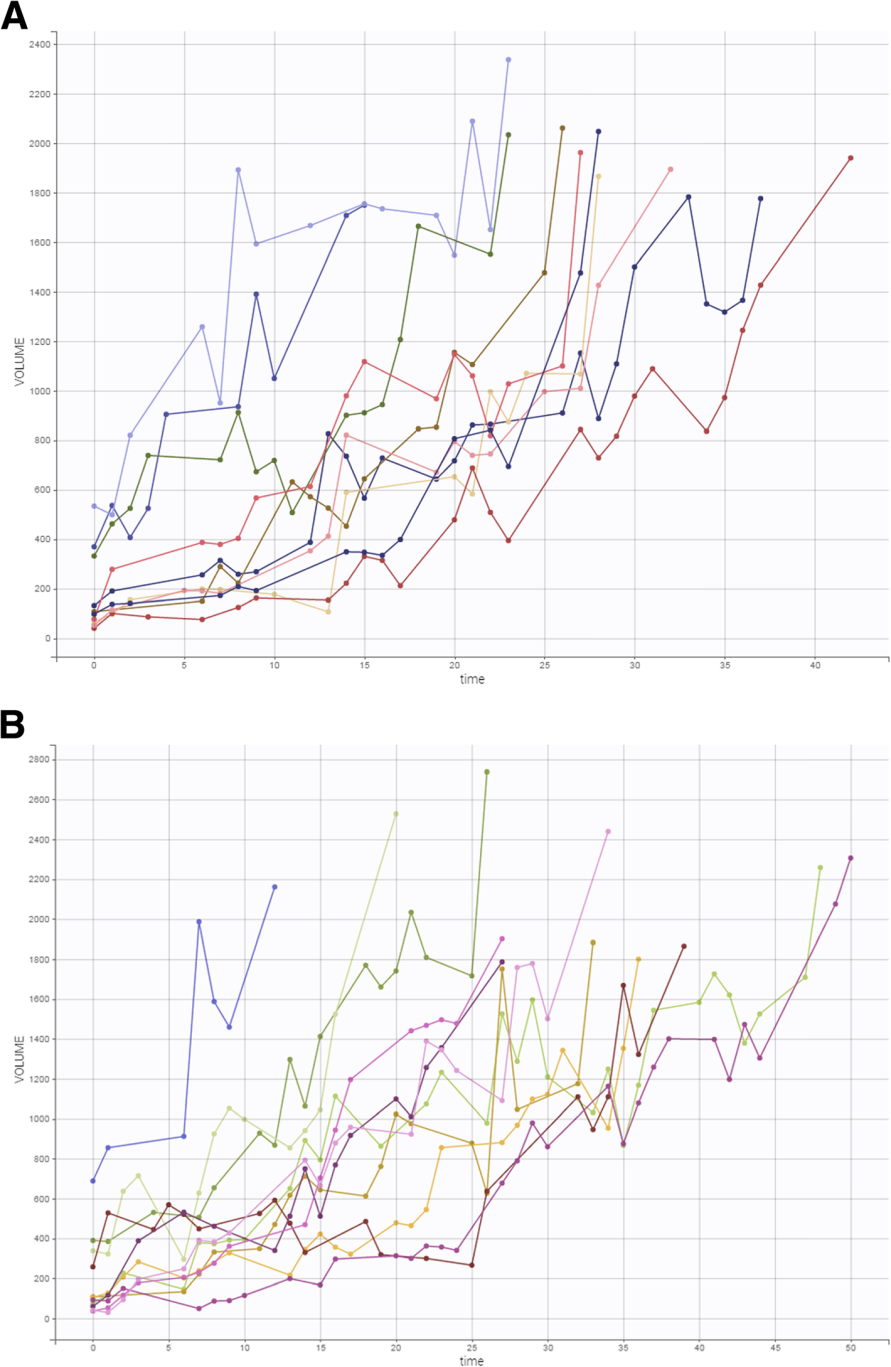
**a**, **b** Time course of tumor growth in 21 untreated tumor-bearing mice over the course of the experiment. The X-axis (time) denotes days, and the Y-axis (volume) denotes mm^3^

In the experiment outlined above, a total of 21 tumor-bearing mice were untreated. Tumor growth over time in this group of animals is shown in Fig. 3.

We use this cohort of controls to investigate various tumor growth functions, namely, generalized logistic, Gompertz, Von Bertalanffny, and Simeoni. We present summary data relative to these fits in Table 1. It turns out that the Simeoni tumor growth function provides the best fit to these data, with minimal AIC, AIC_c_, and BIC. The weights convey the overwhelming preference for the Simeoni tumor growth function among the candidate functions.

**Table 1:** Summary statistics relating to growth curve models fit to the control data

model	− 2*LL	AIC	w(AIC)	AIC_**c**_	w(AIC_**c**_)	BIC	w(BIC)
generalized logistic	5159.69	5179.69	.0037	5180.07	.0038	5190.13	.0038
Gompertz	5182.79	5198.79	2.66E-07	5199.01	2.88E-07	5207.15	7.72E-07
von Bertalanffy	5189.97	5209.97	9.94E-10	5210.35	9.95E-10	5226.42	5.05E-11
Simeoni	5148.52	5168.52	.9963	5168.90	.9963	5179.01	.9962

Notes

*LL* log likelihood, *AIC* Akaike information criterion, *w(AIC)* weights derived from candidate model AIC values, *AIC*_*c*_ corrected Akaike information criterion, *w(AIC*_*c*_*)* weights derived from candidate model AIC_c_ values, *BIC* Bayesian information criteron, *w(BIC)* weights derived from candidate model BIC values

**Fig. 4:**
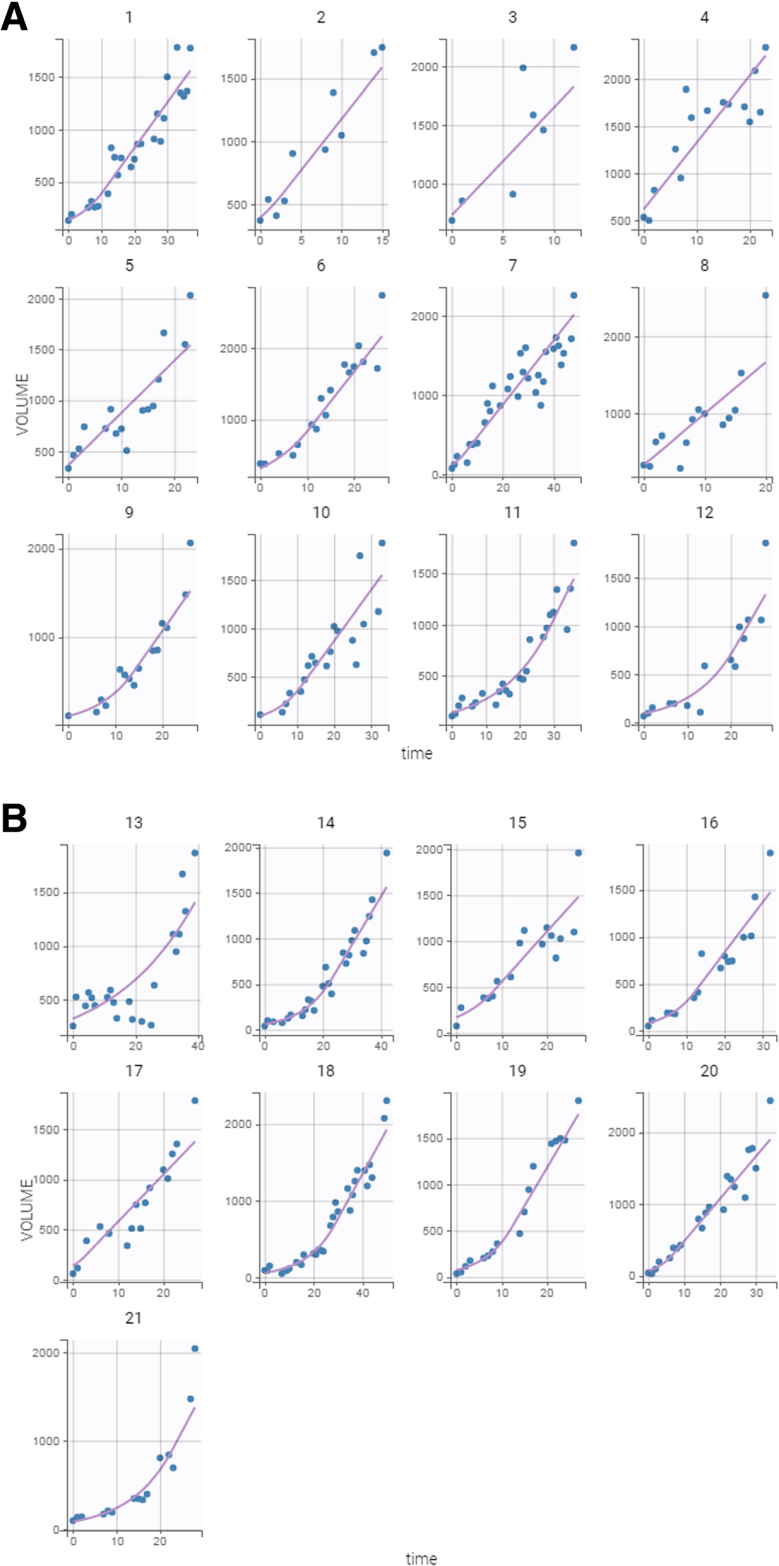
Observed tumor sizes and fitted values of the 21 untreated tumor-bearing mice over the course of the experiment. The Simeoni tumor growth function was fit to the tumor size data from the entire cohort of animals, and individual fits were then derived from the mixed model analysis undertaken in Monolix

Plots of the individual fits with the Simeoni tumor growth function are shown in Fig. 4. Although the individual growth curves are quite heterogeneous (Fig. 3), the fitted curves quite nicely represent the observed data.

**Fig. 5:**
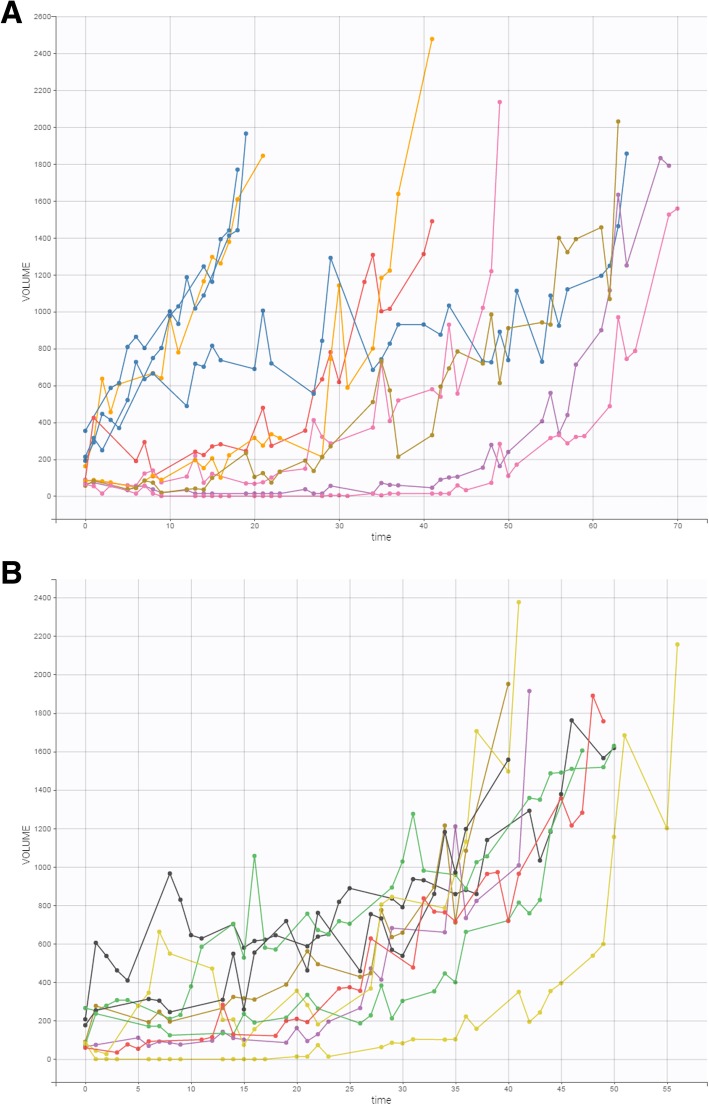
**a**, **b** Time course of tumor growth in 19 treated tumorbearing mice over the course of the experiment. Treatment consisted of a single dose of cisplatin (5 mg/kg) on day 0. The X-axis (time) denotes days, and the Y-axis (volume) denotes mm^3^

Nineteen tumor-bearing mice were treated with cisplatin. Their individual growth patterns are shown in Fig. 5.

We used the Simeoni tumor growth function, and proceeded to fit the Simeoni model with varying numbers of peripheral delay compartments as well as the delay model to these data. Summary statistics are given in Table 2. Among the candidate models, the delay model evinces minimal AIC and AIC_c_ values, whereas the Simeoni model with 2 delay compartments minimizes the BIC. The weights reflect this model uncertainty: one might deem the delay model preferable to the Simeoni - 2 and Simeoni - 3 models relative to AIC or AIC_c_, but this order is reversed with BIC. Regardless, the Simeoni - 1 model appears inferior to the other candidate models.

**Table 2:** Summary statistics relating to growth curves models fit to the treated data

model	-2*LL	AIC	w(AIC)	AIC_**c**_	w(AIC_**c**_)	BIC	w(BIC)
Simeoni -1	6786.12	6818.12	.015	6818.91	.016	6833.23	.020
Simeoni - 2	6780.10	6812.10	.310	6812.89	.325	6827.21	.410
Simeoni - 3	6780.32	6812.32	.278	6813.11	.291	6827.44	.366
delay	6775.61	6811.61	.397	6812.64	.368	6828.61	.204

Notes

1, 2, and 3 in the Simeoni model designations refer to the number of delay compartments incorporated into these models (Fig. 1). The delay model has one peripheral compartment, with an explicit delay in elimination (Fig. 2)

*LL* log likelihood, *AIC* Akaike information criterion, *w(AIC)* weights derived from candidate model AIC values, *AIC*_*c*_ corrected Akaike information criterion, *w(AIC*_*c*_*)* weights derived from candidate model AIC_c_ values, *BIC* Bayesian information criteron, *w(BIC)* weights derived from candidate model BIC values

**Fig. 6:**
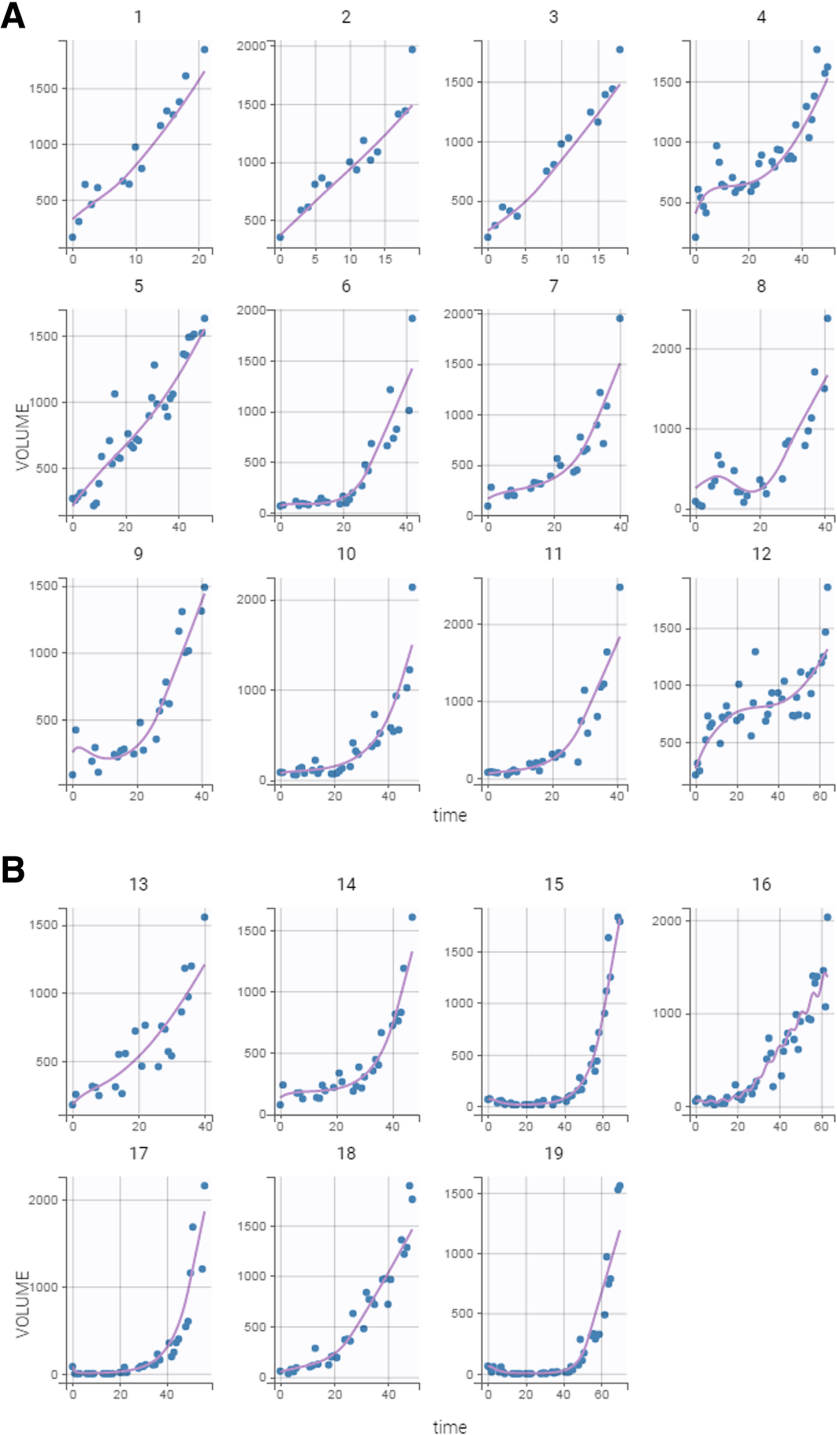
Observed tumor sizes and fitted values of the 19 treated tumor-bearing mice over the course of the experiment. A system of delay differential equations incorporating the Simeoni tumor growth function was fit to the tumor size data from the entire cohort of animals, and individual fits were then derived from the mixed model analysis undertaken in Monolix

Plots of the individual fits with the delay model are shown in Fig. 6. Again, inter-animal variability is quite high, especially in terms of tumor response to treatment, with the individual fits modeling the observed data appropriately.

## Discussion

The Simeoni tumor growth function switches between exponential and linear growth, but without a plateau phase, in contrast to the classic tumor growth functions. It works quite well in our experimental setting, in which tumor-bearing mice were euthanized for ethical reasons when tumor volume reached the maximum allowable tumor mass, and this generally occurred before a plateau phase was reached. We caution, however, that in other experimental or clinical settings, the Simeoni tumor growth function might be supplanted by a more biologically realistic growth law [10, 18]. And, as a reviewer has pointed out, even the classical growth curve models logistic, Gompertz, and von Bertalanffy (our eqs. 5–7) enjoy many variations, specializations, and extensions, which might be judiciously employed to good effect in experimental settings.

The original Simeoni model essentially is a transit compartment model: tumor cells pass through progressive stages of damage because of the drug’s mechanism of action. Dying tumor cells stop proliferating and pass through several stages of dying (depicted by n delay compartments) before eventual death. However, there is some ambiguity over the exact number of delay compartments. With our experimental data, the appropriate number of delay compartments remains somewhat uncertain, relative to goodness of fit. Moreover, we have found that the delay compartments can effectively be replaced by a model incorporating a single peripheral compartment with a delay in elimination; that is, explicit incorporation of a delay term in the Simeoni tumor growth model can operationally replace the multiple transit compartments originally proposed, and effectively model the duration of the death process subsequent to drug action. Delay differential equation models have often been successfully investigated in tumor growth studies [19–24], and seem especially applicable in settings where tumor volume decrease appears delayed with respect to observed drug concentrations. With agents such as cisplatin, the delay in cell kill relative to the time course of extracellular exposure likely reflects the kinetics of cellular drug uptake and binding to intracellular targets [25]. Alternatively, more physiologically based models can ameliorate the ambiguity inherent with an arbitrary number of transit compartments [25–28].

With regard to model selection, we are more interested in the relative performances of the models rather than their absolute AIC, AIC_c_, or BIC values. In this regard, the weights derived from the information criterion values quantify the probabilities of each model being optimal, given the data and the set of candidate models, hence provide useful criteria for model comparisons. There is little uncertainty about the suitability of the Simeoni growth function in Table 1, whereas the identifiability of the best approximating model in Table 2 is not incontrovertible. Regardless, the delay model is certainly competitive with the Simeoni model, and does provide excellent agreement with the experimental data, even with substantial tumor growth heterogeneity and inter-animal diversity in response.

## Conclusions

One should exercise caution if asserting a particular mathematical model uniquely characterizes tumor growth curve data. The Simeoni ODE model of tumor growth is not unique in that alternative models can provide equivalent representations of tumor growth.

## Data Availability

The datasets analysed in this study are available from the corresponding author on reasonable request.

## Abbreviations

AIC: Akaike information criterion
AIC_c_: Corrected Akaike information criterion
BIC: Bayesian information criterion
nlme: Nonlinear mixes effects
ODEs: Ordinary differential equations
pk/pd.: Pharmacokinetic/pharmacodynamic
PRISM: Proteogenomics Research Institute for Systems Medicine
SAEM: Stochastic approximation expectation maximization
